# Resolving the apparent transmission paradox of African sleeping sickness

**DOI:** 10.1371/journal.pbio.3000105

**Published:** 2019-01-11

**Authors:** Paul Capewell, Katie Atkins, William Weir, Vincent Jamonneau, Mamadou Camara, Caroline Clucas, Nono-Raymond K. Swar, Dieudonne M. Ngoyi, Brice Rotureau, Paul Garside, Alison P. Galvani, Bruno Bucheton, Annette MacLeod

**Affiliations:** 1 Wellcome Centre for Molecular Parasitology, College of Medical, Veterinary and Life Sciences, Glasgow Biomedical Research Centre, University of Glasgow, Glasgow, United Kingdom; 2 Department of Infectious Disease Epidemiology, Faculty of Epidemiology and Population Health, London School of Hygiene and Tropical Medicine, London, United Kingdom; 3 Centre for Mathematical Modelling of Infectious Diseases, London School of Hygiene and Tropical Medicine, London, United Kingdom; 4 Centre for Global Health, Usher Institute for Population Health Sciences and Bioinformatics, University of Edinburgh, Edinburgh, United Kingdom; 5 School of Veterinary Medicine, University of Glasgow, Glasgow, United Kingdom; 6 Unité Mixte de Recherche IRD-CIRAD 177, INTERTRYP, Institut de Recherche pour le Développement (IRD), Montpellier, France; 7 Programme National de Lutte contre la Trypanosomiase Humaine Africaine, Conakry, Guinea; 8 University of Kinshasa, Kinshasa, Democratic Republic of the Congo; 9 Trypanosome Transmission Group, Trypanosome Cell Biology Unit, INSERM U1201 and Department of Parasites and Insect Vectors, Institut Pasteur, Paris, France; 10 Center for Infectious Disease Modeling and Analysis, Yale School of Public Health, New Haven, Connecticut, United States of America; Imperial College London, UNITED KINGDOM

## Abstract

Human African trypanosomiasis (HAT), or African sleeping sickness, is a fatal disease found throughout sub-Saharan Africa. The disease is close to elimination in many areas, although it was similarly close to elimination once before and subsequently reemerged, despite seemingly low rates of transmission. Determining how these foci persisted and overcame an apparent transmission paradox is key to finally eliminating HAT. By assessing clinical, laboratory, and mathematical data, we propose that asymptomatic infections contribute to transmission through the presence of an overlooked reservoir of skin-dwelling parasites. Our assessment suggests that a combination of asymptomatic and parasitaemic cases is sufficient to maintain transmission at foci without animal reservoirs, and we argue that the current policy not to treat asymptomatic HAT should be reconsidered.

## Abstract 2

African sleeping sickness is an important disease of sub-Saharan Africa that is approaching elimination, but this Essay maintains that an overlooked anatomical reservoir — human skin — may impact control efforts.

## Introduction

Human African trypanosomiasis (HAT) is a deadly disease of sub-Saharan Africa caused by two morphologically identical subspecies of the vector-borne parasite *Trypanosoma brucei*, *T*. *b*. *rhodesiense* and *T*. *b*. *gambiense*. *T*. *b*. *rhodesiense* causes a more acute form of HAT and is found predominantly in eastern Africa. *T*. *b*. *gambiense* is prevalent in western and central Africa and leads to a more chronic disease. Although there has been a recent resurgence in both forms of HAT, *T*. *b*. *gambiense* is by far the most common and represents over 97% of reported cases [1]. Both human infective forms are transmitted by tsetse flies (*Glossina* spp.) and contribute to a cycle of poverty in some of the poorest regions of sub-Saharan Africa, predominantly in *T*. *b*. *gambiense* endemic areas. HAT is one of the diseases targeted by the WHO 2020 and 2030 elimination goals and, as a result of concerted control measures, the number of annual cases dropped from 22,800 reported (300,000 estimated) cases in 1995 to less than 3,000 reported cases in 2015 and is now approaching elimination in many countries [2]. The disease was similarly close to elimination during the 1960s when intensive control efforts reduced the number of reported cases to just 5,000. Thereafter, political turmoil, coupled with a decline in screening and control programmes, led to a rapid rise and reemergence of several high-prevalence foci, with reported cases reaching a new peak by the end of the 20th century [2]. Renewed HAT control and surveillance efforts through bilateral cooperation between WHO, various nongovernment organisations (NGOs), and African administrations over the last two decades have again presented an opportunity to finally eliminate this debilitating and deadly disease [2]. However, success depends on understanding the mechanisms that led to the persistence and reemergence of HAT in low-transmission settings.

## The transmission paradox

A key issue is the apparent transmission paradox that surrounds the disease as it approaches elimination, particularly with regards *T*. *b*. *gambiense*. Although *T*. *b*. *rhodesiense* often presents with a high parasitaemia, there are relatively few parasites observed in a typical *T*. *b*. *gambiense* infection. Combined with the small volume of a tsetse blood meal (approximately 10 to 30 μL) that limits the opportunity to ingest a parasite, the difficulty in establishing an infection in the tsetse and the low prevalence of infected vectors at many foci [3], it would seem *T*. *b*. *gambiense* transmission should have ceased. These issues lead to the obvious questions: how does *T*. *b*. *gambiense* HAT persist, and how did it return from apparent elimination? Several hypotheses to explain the persistence and reemergence of *T*. *b*. *gambiense* HAT foci have been proposed, including animal reservoirs, the use of suboptimal diagnostic methods, underreporting, and the possible involvement of infectious but persistently asymptomatic individuals [4]. Although trypanosome-infected animals have been detected in many HAT foci, and it is generally accepted that such reservoirs contribute to the maintenance and transmission of *T*. *b*. *rhodesiense*, this is not universally the case for *T*. *b*. *gambiense*. The role that animal reservoirs may play in *T*. *b*. *gambiense* HAT foci persistence has recently been reviewed, suggesting that they contribute to transmission maintenance in some areas [5]. However, there are *T*. *b*. *gambiense* HAT foci that have few or no infected domestic animals despite intensive sampling, indicating that the disease could be maintained by predominantly human infections (or an as yet unidentified wild animal reservoir) [3]. Recently, evidence has also emerged to suggest that aparasitaemic asymptomatic human infections may constitute an overlooked reservoir of disease that contributes to the maintenance of disease foci [6].

## Resolving the paradox

Counter to the long-standing belief that HAT is always fatal in the absence of treatment, recent studies suggest that some individuals infected with *T*. *b*. *gambiense* are able to tolerate the parasite with few specific symptoms [7]. This has not yet been demonstrated in *T*. *b*. *rhodesiense*. These asymptomatic individuals infected with *T*. *b*. *gambiense* are serologically positive but microscopy negative when their blood is examined, even when assayed via the trypanolysis (TL) test that eliminates false positives [8]. In some individuals, lymph node aspirate was also examined and was similarly negative. Current diagnostic protocols for definitive diagnosis of both *T*. *b*. *rhodesiense* and *T*. *b*. *gambiense* HAT rely on the visible identification of parasites in the blood or lymph node aspirate. As asymptomatic infected individuals in *T*. *b*. *gambiense* areas do not present with disease symptoms and have undetectable parasitaemia, they are not diagnosed via passive surveillance, and their potential impact on transmission is overlooked. Even under active surveillance schemes, serologically positive but aparasitaemic and asymptomatic individuals are not treated due to the toxicity of available drugs (pentamidine and eflornithine), potentially meaning they continue to contribute to transmission.

**Fig 1:**
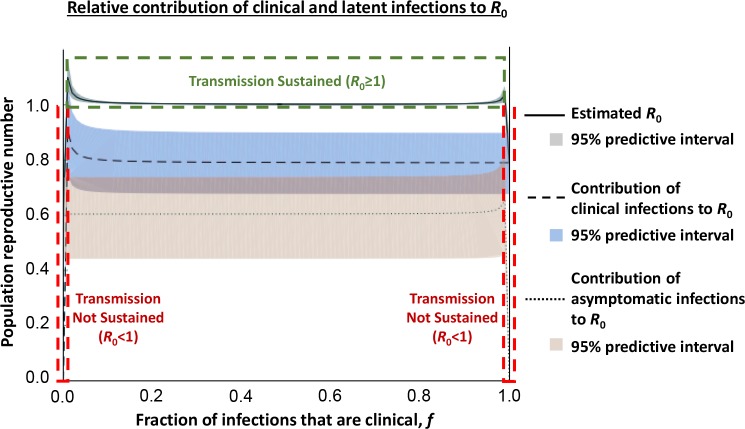
Modelling of the relative contributions of asymptomatic and clinical cases to *R*_0_ in a polymorphic human HAT focus without animal reservoirs. We estimated the contribution of asymptomatic and clinical infections to *R*_0_ (the basic reproductive number) under equilibrium prevalence by adapting a previously published trypanosomiasis transmission model [11]. Consistent with empirical data showing no domestic or wildlife reservoir, we removed the nonhuman-animal contribution to *R*_0_ and instead allowed for a polymorphic human population in which a fraction (*f*) of the population develop clinical infections when infected (population I), with the remainder (1–*f*) developing asymptomatic infections (population II). This leads to $R_{0}=\sqrt{\frac{1}{\left(1-i_{v}^{*})(I_{I}^{*}+I_{II}^{*}\right)}[\frac{I_{I}^{*}}{1-\frac{I_{I}^{*}}{fN}}+\frac{I_{II}^{*}}{1-\frac{I_{II}^{*}}{(1-f)N}}]}$, where i*_v_ is the infected tsetse prevalence, *I*_I_* is the number of clinical infections present at equilibrium, *I*_II_* is the number of asymptomatic infections at equilibrium, and *N* is the total human population size. We simulated this equation with epidemiologic and demographic surveys from the Forécariah focus in Guinea. Specifically, there were 13 clinical infections and 16 suspected asymptomatic infections identified during the survey. Of the suspected asymptomatic individuals, one-third tested negative on follow-up using the TL test and one-third developed symptoms [10]. Therefore, we set the number of asymptomatic infections as *I*_II_* = 16*X* (where *X*~Uniform(1/3,1)) and the number of clinical infections as *I*_I_* = 29–*I*_II_*. The total population size was set as *N* = 10,837, based on 7,586 surveyed individuals and a survey completeness of 70%. These results suggest that transmission is not sustainable (*R*_0_ < 1) when nearly all infections are either clinical (*f* > 0.98) or asymptomatic infections (*f* < 0.02). The shaded areas represent 95% predictive intervals when the number of asymptomatic and clinical infections were sampled 1,000 times. HAT, human African trypanosomiasis; TL, trypanolysis.

This asymptomatic infection period can be extremely protracted, if not indefinite, with a recent case persisting for 29 years before the patient developed symptoms [9]. The ability to occasionally detect parasite genetic material using PCR from these individuals supports the assertion that they harbour active infections, and they have consequently received increased attention in recent years. For example, over a two-year follow-up in the Forécariah focus in Guinea, approximately 13% of serologically positive individuals without visible parasites in an initial screen developed clinical symptoms and parasitaemia [10], suggesting that these individuals were early-stage clinical cases or had been subsequently reinfected by a tsetse fly. Of the remaining positive cases, 39% became serologically negative (indicating clearance of infection or an initial false positive serology result), whereas 48% remained seropositive for at least two years [10], consistent with harbouring latent *T*. *b*. *gambiense* infections. As previously mentioned, few infected animals have been identified in similar settings in Guinea, suggesting that transmission is primarily human to human [3]. Although it may be possible that asymptomatic animal reservoirs harbouring parasites exist, molecular and serological techniques used to identify human asymptomatic cases would also have detected asymptomatic animals, suggesting that there are few such infected animals in these *T*. *b*. *gambiense* foci. Although transmission in this focus appears to be largely human to human, it is unclear what contribution asymptomatic human infections may make to transmission. To address this, we adapted a previously published mathematical model [11] and parameterised it using data from the Forécariah focus [10] to simulate a polymorphic human population (Fig 1). This analysis suggests that both asymptomatic and clinical cases are required to maintain HAT in the absence of animal reservoirs and indicates that long-term asymptomatic human infections may indeed represent an important but overlooked source of transmission. In order to detect both symptomatic and asymptomatic infected individuals, active rather than passive surveillance is required for HAT monitoring. As the number of symptomatic cases falls, the proportion of infected individuals that harbour asymptomatic infections will consequently rise, representing a new challenge to elimination.

## The skin as a reservoir

There are several lines of evidence supporting the suggestion that asymptomatic individuals harbour trypanosomes in their skin and thus form an anatomical reservoir for *T*. *b*. *gambiense*, including xenodiagnosis assays showing that asymptomatic and symptomatic infected humans and animals are able to infect tsetse flies [12,13]. These experiments provide independent validation that tsetse flies can become infected despite feeding on hosts with undetectable parasitaemia, indicating that the parasites are derived from an extravascular location. We hypothesise that the skin is a likely source of these infective trypanosomes due to experimental work in mice, indicating that animals with no visible parasitaemia have extravascular parasites in their dermis and are infectious to the tsetse fly vector [6,14]. The presence of *T*. *brucei* parasites outside the vasculature of the host was recognised in the original characterisation of the disease, although this understanding has been gradually eclipsed by descriptions of *T*. *brucei* as largely a blood parasite. This is in part due to a reliance on the detection of blood parasites for diagnosis.

Recent developments have begun to refocus attention on the existence and significance of extravascular skin-dwelling parasites. These include the description of a *T*. *brucei* adipose tissue form (ATF) in mice, a distinct life-cycle stage of the parasite that exploits fatty acid oxidation to survive in the lipid-rich environment of host adipose tissue [15]. The high density of adipose tissue in the skin would make this a likely site for these forms, although there is not yet any direct evidence of ATF parasites in the skin. Independent experiments have also shown that trypanosomes recently inoculated into mouse skin by tsetse flies reside close to dermal adipocytes and can be retransmitted [14]. Immunohistological analysis of archived human skin punches has found trypanosomes in the skin of individuals not previously diagnosed with HAT, consistent with the hypothesis that these are asymptomatic infections with transmissible extravascular parasites [6]. The hypothesis that asymptomatic individuals harbour skin-dwelling trypanosomes is currently being formally tested in an immunohistological survey of skin biopsies from infected individuals in Guinea.

Given the mean unstressed intravascular volume of the blood (3.8 L) and the average volume of the skin (3.6 L), skin-dwelling parasites may represent a significant population of dividing parasites in a chronic HAT infection that is similar in size to the population of blood parasites. The strategy of residing in the skin may have evolved to maximise passage by telmophagus (slash and suck) insects, like tsetse flies, that rupture the skin rather than feeding on blood directly from active vessels. Indeed, other parasites that exist in the skin of the host, such as *Leishmania* and *Onchocerca*, rely on telmophagus vectors for transmission.

## Future perspectives

There is, therefore, clear evidence from both human and animal studies that skin invasion occurs during *T*. *brucei* infection and likely represents an anatomical reservoir that could resolve the transmission paradox of *T*. *b*. *gambiense* transmission and maintenance at foci without animal reservoirs. The hypothesis that *T*. *b*. *gambiense* infection can lead to the asymptomatic carriage of transmissible parasites has important implications for the WHO 2020 and 2030 HAT elimination goals [2]. Current policy does not advise the treatment of individuals without confirmation of parasites in bodily fluids due to the toxic nature of currently available drugs. However, identification and treatment of asymptomatic infections could improve the effectiveness of disease control approaches and increase the likelihood of HAT elimination at persistent foci. Such efforts could be bolstered by vector-control measures aimed at decreasing human and tsetse contact, with prior studies demonstrating that such efforts [16] reduced *T*. *b*. *gambiense* transmission [17]. Although it would not be advisable for asymptomatic individuals to be treated with currently available drugs due to their toxicity, the development of less harmful alternatives and improving the identification of asymptomatic individuals may remove this barrier. For example, oral fexinidazole and acoziborole have both been shown to be almost as effective as current treatments but with much less toxicity [18,19]. However, it is essential that the capacities of these new drugs to treat skin-dwelling parasites are also ascertained, although only in-hospital and under strictly controlled administration. If efficacy is demonstrated, we argue that there should be a reassessment of the policy not to treat asymptomatic individuals, in addition to concerted surveillance efforts to improve the diagnosis and identification of such individuals. This would potentially remove an important reservoir for *T*. *b*. *gambiense* HAT in many areas, reducing transmission and contributing to the elimination of this devasting disease.

## Abbreviations

ATF: adipose tissue form
HAT: human African trypanosomiasis
NGO: nongovernment organisation
TL: trypanolysis
